# Arterial Hypertension and Tyrosine Kinase Inhibitors in Chronic Myeloid Leukemia: A Systematic Review and Meta-Analysis

**DOI:** 10.3389/fphar.2021.674748

**Published:** 2021-09-22

**Authors:** Olga Mulas, Giovanni Caocci, Brunella Mola, Giorgio La Nasa

**Affiliations:** Hematology Unit, Businco Hospital, Department of Medical Sciences and Public Health, University of Cagliari, Cagliari, Italy

**Keywords:** chronic myeloid, leukemia, tyrosine kinase inhibitor, hypertension, cardiovascular

## Abstract

**Background:** Off-target effects in chronic myeloid leukemia (CML) patients treated with tyrosine kinase inhibitors (TKIs) are associated with cardiovascular toxicity. Hypertension represents an important cardiovascular complication and, if not appropriately managed, can contribute to developing thrombotic events. Third-generation TKI ponatinib is associated with hypertension development, and its use is more restricted than in the past. Few data are reported for second-generation TKI, nilotinib, dasatinib, and bosutinib. The aim of this article was to evaluate with a systematic review and meta-analysis the real incidence of hypertension in CML patients treated with second- or third-generation TKI.

**Methods:** The PubMed database, Web of Science, Scopus, and ClinicalTrials.gov were systematically searched for studies published between January 1, 2000, and January 30, 2021; the following terms were entered in the database queries: Cardiovascular, Chronic Myeloid Leukemia, CML, Tyrosine kinases inhibitor, TKI, and Hypertension. The study was carried out according to the Preferred Reporting Items for Systematic and Meta-Analyses (PRISMA) statement.

**Results:** A pooled analysis of hypertension incidence was 10% for all new-generation TKI, with an even higher prevalence with ponatinib (17%). The comparison with the first-generation imatinib confirmed that nilotinib was associated with a significantly increased risk of hypertension (RR 2; 95% CI; 1.39-2.88, I^2^=0%, z=3.73, p=0.0002). The greatest risk was found with ponatinib (RR 9.21; 95% CI; 2.86-29.66, z=3.72, p=0.0002).

**Conclusion:** Hypertension is a common cardiovascular complication in CML patients treated with second- or third-generation TKI.

## Introduction

Chronic myeloid leukemia (CML) is a hematological disease characterized by the uncontrolled proliferation of hematopoietic stem cells due to a characteristic genetic anomaly causing the synthesis of the abnormal protein Bcl-Abl1 (Faderl et al., 1999). Tyrosine kinase inhibitors (TKIs) specifically targeting the Bcl-Abl1 protein have been developed, resulting in a dramatic change in the prognosis of the disease (Hochhaus et al., 2017). Nowadays, several molecules have emerged, together with imatinib, in the treatment of CML (Cortes et al., 2016a; Hochhaus et al., 2016a; Cortes et al., 2018a; Cortes et al., 2018b). Second- and third-generation TKI can provide faster molecular responses but are considered less safe than first-generation drugs. Although all second-generation TKIs can be used as first-line treatments, evidence-based guidelines recommend taking into account target profundity of molecular response and TKI safety profiles for the final treatment decision (Fachi et al., 2018; Haguet et al., 2020). The use of a second-generation TKI over imatinib is particularly recommended for patients with moderate- or high-risk Sokal scores. Second-generation TKIs are also recommended for younger patients because of the higher probability of treatment-free remission with these TKIs (Deininger et al., 2020; Hochhaus et al., 2020). Due to the growing number of long-surviving patients who undergo TKI treatment for many years, the problem of long-term toxicities has emerged (Steegmann et al., 2016). Cardiovascular (CV) toxicity has a potentially important impact on long-term morbidity and mortality in these patients. Particularly, nilotinib and third-generation TKI ponatinib are more frequently associated with the onset of cardiovascular events, especially thrombotic events (Aghel et al., 2017). Hypertension represents, per se, a comorbidity that can increase the CV risk of patients (Piepoli et al., 2016). Exacerbation of hypertension and an increase of new events were reported, especially with the use of ponatinib since its pivotal trials (Cortes et al., 2013; Lipton et al., 2016). Since then, many studies have highlighted its cardiotoxic profile and the possible mechanism (Valent et al., 2017). Currently, limited use of ponatinib in those patients who already have cardiovascular comorbidities has been recommended (Deininger et al., 2020). In contrast, second-generation TKIs, such as dasatinib and bosutinib, seem to be safer (Medeiros et al., 2018).

The aim of this systematic review and meta-analysis has been to evaluate the real incidence of hypertension, considering also the real-life data in CML patients treated with new-generation TKI (NGTKI).

## Materials and Methods

### Search Strategy

**FIGURE 1:**
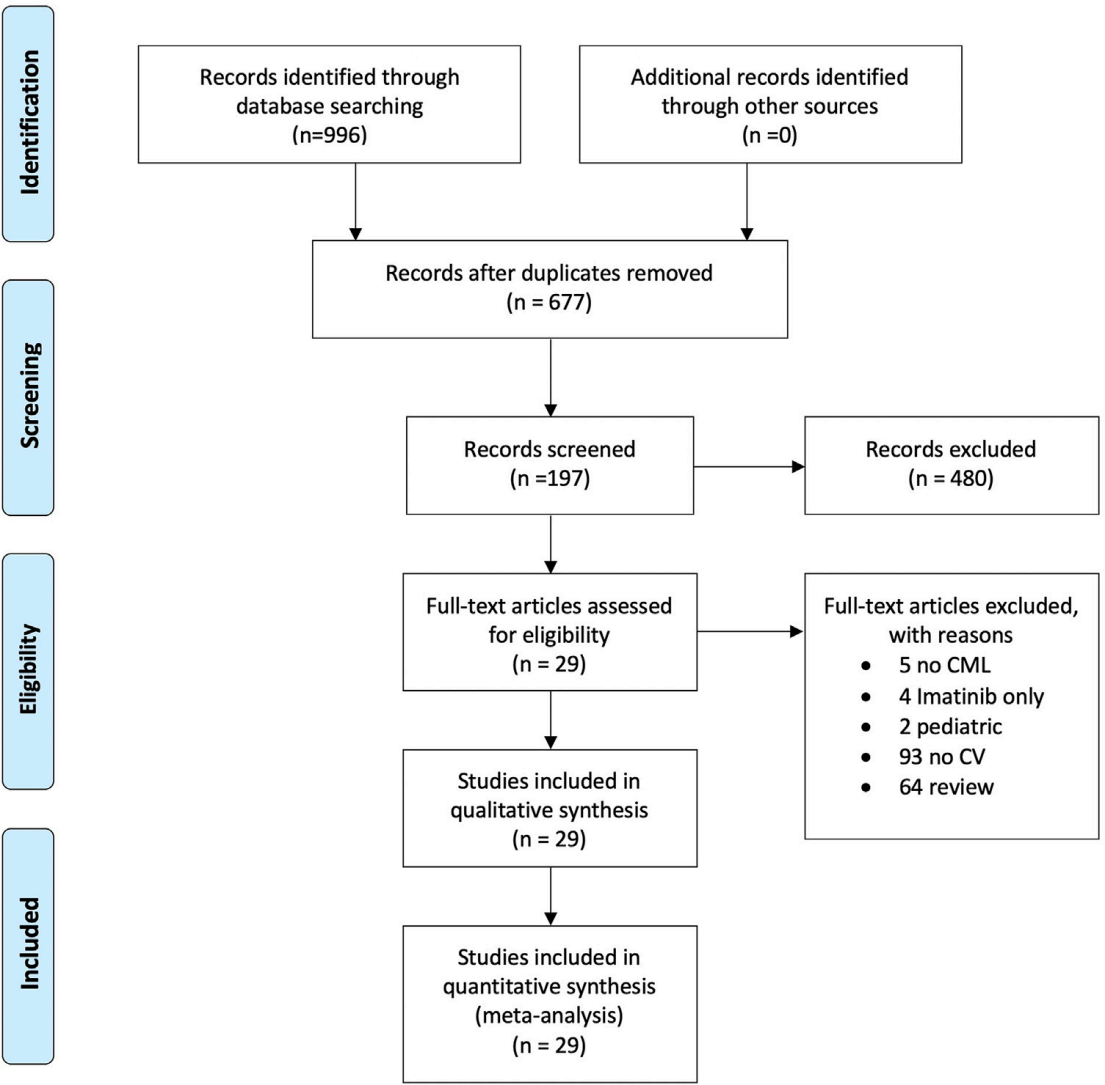
Study flow diagram.

A systematic literature search on PubMed, Web of Sciences, Scopus, and ClinicalTrials.gov was performed to find studies on CML treated with second- or third-generation tyrosine kinase inhibitors and cases of hypertension published from January 1, 2000, to January 30, 2021. Using MeSH headings, we searched for the terms “Chronic Myeloid leukemia,” “CML,” “Tyrosine kinase inhibitors,” “TKI,” “Hypertension,” and “Cardiovascular,” as well as variations thereof. The results were defined using the Preferred Reporting Items for Systematic and Meta-Analyses (PRISMA) statement to identify, select, and determine the eligibility of articles for inclusion in the study. Figure 1 shows the study flow diagram. Quality rating of randomized clinical trials and observational studies was performed using the NIH Study Quality Assessment Tools (Study Quality Assessment, 2020), and the results are shown in Supplementary Table S1. The systematic search strategy is available in Supplementary Table S2.

### Inclusion Criteria

Studies were included in this analysis if they were (Faderl et al., 1999) randomized controlled trials or cohort studies of adult patients of at least 18 years old treated with second- or third-generation TKIs (nilotinib, dasatinib, bosutinib, and ponatinib) for chronic, accelerated, and blastic CML phases (Hochhaus et al., 2017); studies reporting hypertension events (Cortes et al., 2016a); single cohort studies or a comparison study of second- or third-generation TKI versus imatinib (Cortes et al., 2018a); indicating the time of exposure to TKI (Hochhaus et al., 2016a); and in the English language. We included conference abstracts only if they met inclusion criteria and sufficient data were available for the prespecified analysis plan. Finally, for some clinical trials, we used the data found at clinicaltrials.gov because they were complete compared to any otherwise published version.

### Statistical Analysis

Pooled incidence rates of hypertension (including both single- and double-arm studies) were calculated using a single-proportion random-effect model. The analysis was also carried out to evaluate the duration of TKI exposure. The incidence rate allows taking into account sample size and time to exposure in the estimation of the proportion of cases with the predefined outcome. The incidence rate was calculated based on person-time at exposition (Szklo and Nieto, 2007). For studies that compared the rate of hypertension events between two different TKIs, we measured risk ratio with corresponding 95% CI using the DerSimonian and Laird method for the random-effect model. To assess heterogeneity between the studies, the chi-squared test (for evaluation of heterogeneity between studies statistically; *p* less than 0.05) and I^2^ index (to evaluate the heterogeneity of the results) were used with an I^2^ value <25% reflecting mild heterogeneity, 25–50% reflecting moderate heterogeneity, and >50% reflecting severe heterogeneity (Higgins and Thompson, 2002). The analyses were conducted using STATA version 16.1 and Review Manager 5.4.

## Results

Overall, 996 articles were found in the preliminary analysis, and after the subsequent screening, 197 studies were evaluated. Finally, 29 articles were included in the qualitative analysis, with a total sample of 5,533 patients examined. Overall, 29 studies were considered for the quantitative analysis, 28 in the pooled analysis, and 10 in the meta-analysis (Figure 1).

### Quality Assessment

The analysis of the risk of bias is reported in Supplementary Table S1.

In our work, we considered both the retrospective analysis and phase 2 and 3 trials reporting clinical cases of new-onset hypertension during TKI treatment. This choice determines lack of homogeneity in the number and type of previous therapy lines and median exposure times. The number of patients considered in each study is highly variable, ranging from 5 to 1,089 patients, and sample justification is rarely given. In some cases, median exposition time was fairly short, making it difficult to see medium- and long-term adverse effects like the one we are considering. Unfortunately, many of them did not distinguish between different grades of hypertension, only reporting the rough number of cases. This is considered an important bias for the analysis, not allowing a good estimate of the severity of hypertension and its potential clinical outcome.

The majority of the studies analyzed lack the details of the randomization process, the selection of the reported outcome, and the mean dose of the drug used. Many studies allowed dose adjustment because of adverse effects or scarce disease control, making it impossible to clearly define whether dose variation can modify the risk of hypertension. Lastly, consideration of potential confounding variables in the planning of the study is not always performed. Overall, the quality of clinical trials reported is fair or good. Only five studies are considered poor the reasons being that the study population was not clearly defined, or adverse effects and responses were not stratified considering different TKI dosages.

### Qualitative Analysis

Characteristics of studies are available in Table 1. Overall, seven studies were evaluated for bosutinib. The frequency of hypertensive events varied between 2 and 9%. Among these, four were clinical trials considering patients since the second line of treatment (Gambacorti-Passerini et al., 2014; Gambacorti-Passerini et al., 2018; Hino et al., 2020; Pfizer, 2021). In contrast, only two retrospective articles have analyzed patients treated with subsequent lines (Caocci et al., 2019a; García-Gutiérrez et al., 2019). Almost all the detected studies on dasatinib were clinical trials considering patients on the first or second line of treatment (Maiti et al., 2020; Bristol-Myers Squibb, 2016; Bristol-Myers Squibb, 2015 (clinicaltrials, (2021). Im, 2021; Kantarjian et al., 2009). One article evaluated patients retrospectively collected, including the same line of treatment (Suh et al., 2017). The range of events was between 0 and 15%. Similarly, in the nilotinib setting, only one article reported the incidence of hypertension in patients retrospectively evaluated since the second line of treatment (Suh et al., 2017). The other ones were clinical trials evaluating patients in the first or second line of treatment (Hughes et al., 2014; Cortes et al., 2016b; Hochhaus et al., 2016b; Saydam et al., 2016; Novartis Pharmaceuticals, 2020; clinicaltrials (2021). Ph, 2021). The rate of hypertension was higher, between 5 and 19%. On the contrary, the identified articles on ponatinib were mostly retrospective studies (Breccia et al., 2018; Heiblig et al., 2018; Caocci et al., 2019b; Devos et al., 2019; Fava et al., 2019; Binotto et al., 2020; Iurlo et al., 2020), and as expected, they collected data on treatment lines higher than the third. In the clinical trials evaluated, ponatinib was administered as the first- or second-line treatment (Jain et al., 2015; Sanford et al., 2015; Cortes et al., 2018b; clinicaltrials (2021). Po, 2021). In this case, the frequency of hypertension was significantly increased, varying between 2 and 80%.

**TABLE 1:** Characteristics of the studies examined.

Study	Treatment		Number of patients		Line of treatment	Median age, Years		Sex male NGTKI, %	Median time exposure NGTKI, Years	HTN events, (%)	
	Arm 1	Arm 2	Arm 1	Arm 2		Arm 1	Arm 2			Arm 1	Arm 2
Bfore (Pfizer, 2021)	Bosutinib	NA	268	NA	I	52	NA	57,7	2	14 (5)	NA
García-Gutiérrez, 2018 (García-Gutiérrez et al., 2019)	Bosutinib	NA	62	NA	IV	NA	NA	NA	0,76	3 (5)	NA
Hino, 2020 (Hino et al., 2020)	Bosutinib	NA	60	NA	I	55	NA	60	1,4	1 (2)	NA
Gambacorti-Passerini, 2018 (Gambacorti-Passerini et al., 2018)	Bosutinib	NA	284	NA	II	NA	NA	52	2,1	26 (9)	NA
Bela (Gambacorti-Passerini et al., 2014)	Bosutinib	Imatinib	248	251	I	48	47	60	2,5	15 (6)	10 (4)
Caocci, 2019 (Caocci et al., 2019a)	Bosutinib	NA	54	NA	II/III/IV	54	NA	50	1,3	0 (0)	NA
Maiti, 2020 (Maiti et al., 2020)	Dasatinib	NA	149	NA	I	48	NA	58,6	6,5	23 (15)	NA
Dasision, Bristol-Myers Squibb (2016)	Dasatinib	Imatinib	259	260	I	46	49	56	8	26 (10)	20 (8)
S0325 (clinicaltrials (2021). Im, 2021)	Dasatinib	Imatinib	122	123	I	47	50	60	3	1 (1)	0
Suh, 2017 (Suh et al., 2017)	Dasatinib	Nilotinib	81	120	I/II	55	52	70	1,4 (D) / 2 (N)	0 (0)	1
START Rollover Bristol-Myers Squibb (2015)	Dasatinib	Imatinib	185	14	II	NA	NA	50,8	6.8	12 (6)	0
Star-R (Kantarjian et al., 2009)	Dasatinib	Imatinib	101	49	II	51	NA	52	NA	10 (11)	0
ENESTnd (Novartis Pharmaceuticals, 2020)	Nilotinib	Imatinib	563	283	I	NA	NA	58	11	105 (19)	28 (10)
Lasor (Cortes et al., 2016b)	Nilotinib	Imatinib	96	96	II	46	44	56	1,9	5 (5)	2 (2)
Saydam, 2018 (Saydam et al., 2016)	Nilotinib	NA	112	NA	I	47	NA	56,3	2	2 (2)	NA
ENESTcmr (Hughes et al., 2014)	Nilotinib	Imatinib	104	103	II	46	52	68,3	4	10 (10)	6 (6)
NCT00129740 (clinicaltrials (2021). Ph, 2021)	Nilotinib	NA	148	NA	I	51	NA	59,5	11,5	28 (19)	NA
ENEST1st (Hochhaus et al., 2017)	Nilotinib	NA	1089	NA	I/II	53	NA	59	2	65 (6)	NA
Caocci, 2019 (Caocci et al., 2019b)	Ponatinib	NA	85	NA	II/III/IV	53	NA	55	2,3	12 (14)	NA
Devos, 2019 (Devos et al., 2019)	Ponatinib	NA	50	NA	≥II	NA	NA	NA	1	1 (2)	NA
Fava, 2019 (Fava et al., 2019)	Ponatinib	NA	34	NA	II/III/IV	62	NA	50	3,9	3 (9)	NA
Epic (Sanford et al., 2015)	Ponatinib	Imatinib	154	152	I	55	52	63	0,4	28 (18)	3 (2)
Binotto, 2018 (Binotto et al., 2020)	Ponatinib	NA	62	NA	≥II	57,5	NA	53,2	1,8	2 (3)	NA
Heiblig, 2018 (Heiblig et al., 2018)	Ponatinib	NA	62	NA	II/III/IV	47,6	NA	50	1,6	12 (19)	NA
Pace (Cortes et al., 2018b)	Ponatinib	NA	449	NA	≥II	59	NA	53	4,7	142 (32)	NA
Breccia, 2018 (Breccia et al., 2018)	Ponatinib	NA	29	NA	II	54	NA	58,6	1	3 (10)	NA
NCT01570868 (Jain et al., 2015)	Ponatinib	NA	51	NA	I	43	NA	52,9	2,5	15 (29)	NA
Iurlo, 2020 (Iurlo et al., 2020)	Ponatinib	NA	52	NA	II/III/IV	52,9	NA	53,8	1,6	6 (12)	NA
NCT01746836 (Sanford et al., 2015)	Ponatinib	NA	5	NA	II	50	NA	NA	1,8	4 (80)	NA

### Quantitative Assessment

**FIGURE 2:**
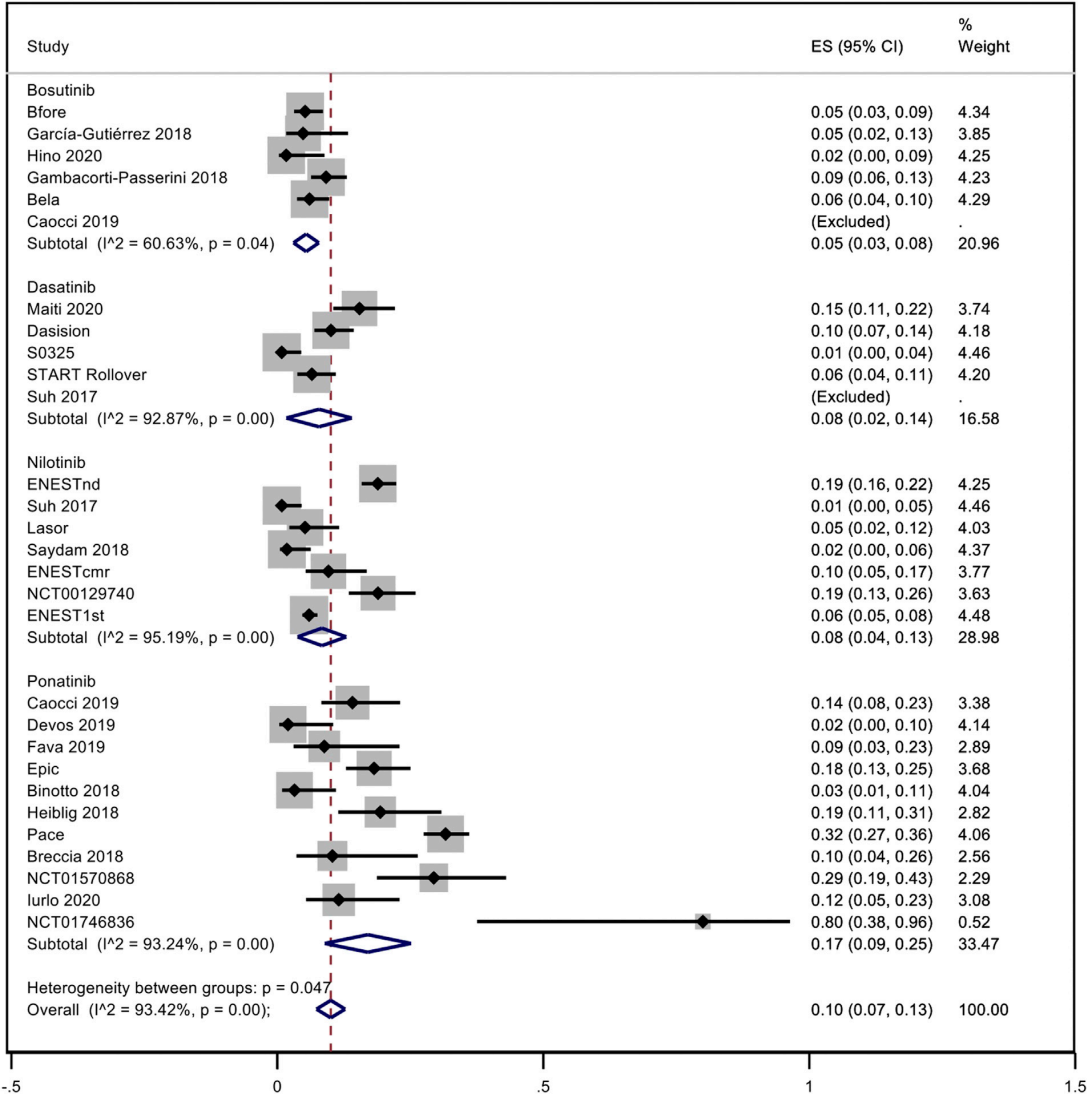
Pooled incidence rate of hypertension in patients treated with second- or third-generation TKI.

**FIGURE 3:**
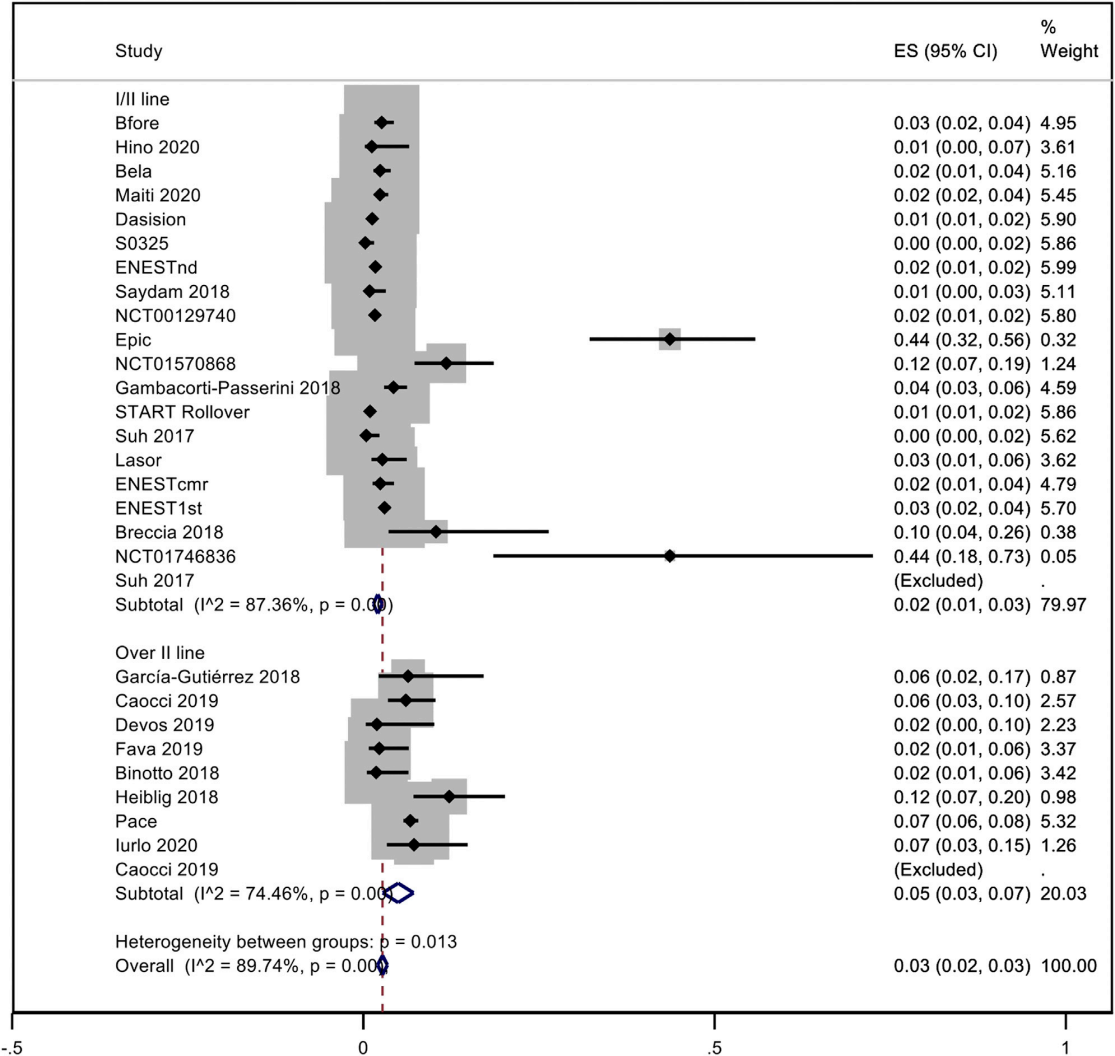
Pooled rate of hypertension when TKI were used in the first- or second-line versus over second-line.

A pooled analysis of the incidence rate of hypertension was carried out considering all the studies with inclusion criteria. Only one study was not included in the analysis because it was not possible to evaluate the time of exposure to dasatinib (Kantarjian et al., 2009). No distinction between observational studies and trials was made. Considering all TKIs, the pooled proportion of hypertension was 10% (95% CI; 0.07–0.13, I^2^ = 93.42%). Subanalysis for each NGTKI showed a pooled rate of 17% (95% CI; 0.09–0.25, I^2^ = 93.24%) for ponatinib, 8% (95% CI; 0.04–0.13, I^2^ = 95.19%) for nilotinib, 8% (95% CI; 0.02–0.14, I^2^ = 92.87%) for dasatinib, and 5% (95% CI; 0.03–0.08, I^2^ = 60.63%) for bosutinib (Figure 2). A further analysis was made to evaluate the pooled rate of hypertension when TKIs were used in the first- or second-line treatment versus over the second-line treatment, showing 9% (95% CI; 0.06–0.12, I^2^ = 92.28%) and 12% (95% CI; 0.03–0.21, I^2^ = 94.61%), respectively (Figure 3). If the analysis was conducted considering the mean exposure time, the pooled proportion of hypertension was 3% (95% CI; 0.02–0.03, I^2^ = 89.74%). In the ponatinib subset, the pooled incidence was 8% (95% CI; 0.05–0.11, I^2^ = 86.80%). A reduction was detected in nilotinib and dasatinib studies, with 2% (95% CI; 0.01–0.02, I^2^ = 76.82%) and 1% (95% CI; 0.00–0.02, I^2^ = 81.41%), respectively. A reduction was observed also for bosutinib with 3% (95% CI; 0.02–0.04, I^2^ = 39.31%) (Supplementary Figure S1). The pooled proportion for lines of treatment subdivision showed a decrease with 2% (95% CI; 0.01–0.03, I^2^ = 87.36%) and 5% (95% CI; 0.03–0.07, I^2^ = 74.46%) in the first or second line versus over second line, respectively (Supplementary Figure S2).

**FIGURE 4:**
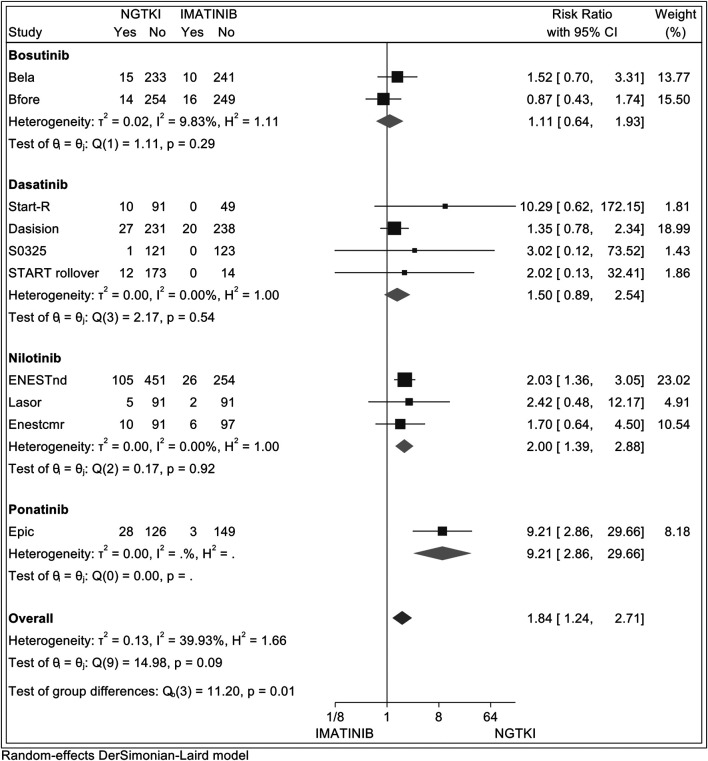
Forest plot showing random-effect meta-analysis of hypertension between imatinib and subsequent-generation TKI.

In addition, a comparative analysis between NGTKI and imatinib was made, with the results shown in Figure 4. Overall, a significantly increased risk of hypertension was detected in NGTKI compared to imatinib, with a risk ratio (RR) of 1.84 (95% CI; 1.24–2.71, I^2^ = 39.93%, z = 3.05, *p* = 0.002). Analysis by the subgroup showed a trend of increased risk of hypertension, without significant results, for bosutinib and dasatinib with an RR of 1.11 (95% CI; 0.64–1.93, I^2^ = 25%, z = 0.39, *p* = 0.70) and 1.50 (95% CI; 0.89–2.54, I^2^ = 0%, z = 1.52, *p* = 0.13), respectively. Nilotinib is associated with an increased significant risk of hypertension with an RR of 2 (95% CI; 1.39–2.88, I^2^ = 0%, z = 3.73, *p* = 0.0002). The greater risk is found with ponatinib RR 9.21 (95% CI; 2.86–29.66, z = 3.72, *p* = 0.0002).

## Discussion

The targeted approach with TKI has revolutionized the treatment of CML, and been able to ensure a life expectancy for patients similar to that of the general population (Bower et al., 2016). Unfortunately, off-target side effects are increasing with the use of these drugs; in particular CV toxicities are leading to significant morbidity and mortality (Damrongwatanasuk and Fradley, 2017). Nowadays, the risk of CV events is well established with nilotinib and ponatinib due to an increase in occlusive events, including myocardial infarction (MI), cerebrovascular accidents (CVAs), and peripheral arterial disease (PAOD) (Medeiros et al., 2018). Hypertension, if not appropriately managed, can be strongly associated with high incidence of CV events (Williams et al., 2018) and can represent a leading cause of CV-related mortality (Lewington et al., 2002). In CML patients, an increase in hypertensive events has been reported with ponatinib, with an incidence of 20–30% in pivotal trials (Lipton et al., 2016; Cortes et al., 2018b). The hypertensive complication is not surprising, given the significant inhibition of ponatinib on vascular endothelial growth factor 2 (VEGFR2) (Ai et al., 2018). VEGF signaling plays a key role in angiogenesis; blocking this pathway not only has antitumor effects but also leads to accelerated hypertension possibly via decreased nitric oxide bioavailability, increased endothelin-1 production, or microvascular rarefaction (Herrmann, 2020). Hypertension is a common event also with nilotinib (Herrmann, 2020). It exerts direct proatherogenic and antiangiogenic effects on vascular endothelial cells, which may contribute to the development of damage in the vascular tissue (Hadzijusufovic et al., 2017). Instead, weak data are available with other TKIs. Another important mechanism associated with hypertension development is the renin–angiotensin system (RAS), which may be found in a circulating form or as a specific tissue expression. Particularly, local bone marrow RAS plays a crucial role in proliferative events, mobilization, angiogenesis, and fibrosis. This has been associated with hypertension development and with atheromatic vascular disease. Furthermore, angiotensin II would appear to favor erythroid proliferation and stimulate differentiation of hematopoietic CD34 progenitors(56). Thus, hypertension and CML could have with RAS an interesting common ground.

Our analysis showed that the pooled hypertension rate of the second- and third-generation TKI is 10%, and it confirms the higher proportion in the ponatinib subgroup. The exposuretime correction shows a reduction in the proportion of hypertension incidence, more evident for dasatinib and nilotinib. A recent analysis of the Food and Drug Administration (FDA) adverse event reporting system database highlighted that ponatinib was the only TKI related to hypertension, with a median time to onset estimated at 53 days (Cirmi et al., 2020). Comparison with first-generation imatinib highlights the increased risk of hypertension events in patients treated with NGTKI, especially with nilotinib and ponatinib. Recently, a real-life monocentric experience showed an increased incidence of cardiovascular events in patients treated with nilotinib and dasatinib compared to the imatinib group, in particular with an increased incidence of hypertension of 7 and 4%, respectively (Novo et al., 2020). Exposure to more than two lines of treatment can be another important element of increased risk in hypertension events. This consideration finds similar results in the incidence of thrombotic events (Chai-Adisaksopha et al., 2016; Caocci et al., 2019c; Caocci et al., 2020). A pooled analysis of major arterial events showed, indeed, a higher rate in patients treated with a subsequent line of TKI compared with those treated with single-line treatment (Chai-Adisaksopha et al., 2016). These findings confirmed that more attention should be given to patients treated with multiple TKI lines. Practical recommendation emphasizes that patients, before starting NGTKI, should be assessed for increased risk of hypertension and associated comorbidities such as cardiovascular disease, diabetes, and kidney disease and for patient characteristics, including race and age. Moreover, early signs of arterial hypertension during TKI treatment should be investigated and treated early. In this context, reaching a treatment-free remission (TFR) could be, therefore, a fair compromise in those patients with high Sokal risk score but an unfavorable cardiovascular profile (Breccia et al., 2020). So far, different treatments are available in the management of hypertension (Abruzzese et al., 2014). The use of dihydropyridine calcium channel blockers and renin–angiotensin system inhibitors (RASi) would be preferable due to the strong selectivity for the vascular compartment (Hayman et al., 2012). In addition, the bone marrow RAS is finely implicated in the development of hypertension (Ciftciler and Haznedaroglu, 2020). In fact, recently, RAS inhibitors (RASi) have been associated with a reduction in CV events in CML patients treated with NGTKI (Mulas et al., 2020). Taken together, the use of RASi could play an important role in these patients.

Our study has some limitations. The principal limitation was the high level of heterogeneity among the studies that did not allow a univocal interpretation of the results. Another limitation was the bias of inclusion criteria in clinical trials, where cardiovascular events were a criterion of exclusion. In addition, data about the time of exposure and patient characteristics were missing in the retrospective studies. In the PACE study, 68% of patients developed increased blood pressure at the 48-month follow-up, with some cases of hypertensive crisis reported. We chose to consider only new diagnosis of hypertension, which was 32% at the 5-year follow-up (Cortes et al., 2018b). In retrospective studies, this distinction was not always possible.

In conclusion, NGTKIs are associated with higher incidence of hypertension. Timely recognition and treatment would allow a reduced risk of developing cardiovascular events.

## Data Availability

The raw data supporting the conclusion of this article will be made available by the authors without undue reservation.
