# Supplementary material for: Arterial Hypertension and Tyrosine Kinase Inhibitors in Chronic Myeloid Leukemia: A Systematic Review and Meta-Analysis
Source: Front Pharmacol. 2021 Sep 22;12:674748. doi: 10.3389/fphar.2021.674748 (PMC8493251; doi:10.3389/fphar.2021.674748)
Supplement: Supplementary file 1 [file Table1.docx]

| Study | Treatment | Quality assessment |
| --- | --- | --- |
|  |  |  |
| Bfore^21^ | Bosutinib | Fair |
| García-Gutiérrez 2018^26^ | Bosutinib | Poor |
| Hino 2020^22^ | Bosutinib | Fair |
| Gambacorti-Passerini 2018^24^ | Bosutinib | Good |
| Bela^23^ | Bosutinib | Fair |
| Caocci 2019^25^ | Bosutinib | Fair |
| Maiti 2020^27^ | Dasatinib | Good |
| Dasision^28^ | Dasatinib | Fair |
| S0325^30^ | Dasatinib | Poor |
| Suh 2017^32^ | Dasatinib | Poor |
| START Rollover^29^ | Dasatinib | Fair |
| Star-R^31^ | Dasatinib | Fair |
| ENESTnd^33^ | Nilotinib | Good |
| Lasor^34^ | Nilotinib | Fair |
| Saydam 2018^35^ | Nilotinib | Fair |
| ENESTcmr^36^ | Nilotinib | Fair |
| NCT00129740^38^ | Nilotinib | Fair |
| ENEST1st^37^ | Nilotinib | Good |
| Caocci 2019^39^ | Ponatinib | Fair |
| Devos 2019^40^ | Ponatinib | Poor |
| Fava 2019^41^ | Ponatinib | Poor |
| Epic^47^ | Ponatinib | Fair |
| Binotto 2018^44^ | Ponatinib | Fair |
| Heiblig 2018^42^ | Ponatinib | Fair |
| Pace^6^ | Ponatinib | Fair |
| Breccia 2018^43^ | Ponatinib | Fair |
| NCT01570868^46^ | Ponatinib | Fair |
| Iurlo 2020^45^ | Ponatinib | Fair |
| NCT01746836^47^ | Ponatinib | Good |

Supplemental table 1
