# Supplementary material for: Arterial Hypertension and Tyrosine Kinase Inhibitors in Chronic Myeloid Leukemia: A Systematic Review and Meta-Analysis
Source: Front Pharmacol. 2021 Sep 22;12:674748. doi: 10.3389/fphar.2021.674748 (PMC8493251; doi:10.3389/fphar.2021.674748)
Supplement: Supplementary file 2 [file Table2.docx]

| Database | Search strategy |
| --- | --- |
| PubMed | ("chronic myeloid leukaemia"[All Fields] OR "leukemia, myelogenous, chronic, bcr abl positive"[MeSH Terms] OR ("leukemia"[All Fields] AND "myelogenous"[All Fields] AND "chronic"[All Fields] AND "bcr abl"[All Fields] AND "positive"[All Fields]) OR "bcr-abl positive chronic myelogenous leukemia"[All Fields] OR ("chronic"[All Fields] AND "myeloid"[All Fields] AND "leukemia"[All Fields]) OR "chronic myeloid leukemia"[All Fields]) AND (("hypertense"[All Fields] OR "hypertension"[MeSH Terms] OR "hypertension"[All Fields] OR "hypertension s"[All Fields] OR "hypertensions"[All Fields] OR "hypertensive"[All Fields] OR "hypertensive s"[All Fields] OR "hypertensives"[All Fields] OR ("cardiovascular system"[MeSH Terms] OR ("cardiovascular"[All Fields] AND "system"[All Fields]) OR "cardiovascular system"[All Fields] OR "cardiovascular"[All Fields] OR "cardiovasculars"[All Fields])) AND ("dasatinib"[MeSH Terms] OR "dasatinib"[All Fields] OR ("4 methyl n 3 4 methylimidazol 1 yl 5 trifluoromethyl phenyl 3 4 pyridin 3 ylpyrimidin 2 yl amino benzamide"[Supplementary Concept] OR "4 methyl n 3 4 methylimidazol 1 yl 5 trifluoromethyl phenyl 3 4 pyridin 3 ylpyrimidin 2 yl amino benzamide"[All Fields] OR "nilotinib"[All Fields]) OR ("bosutinib"[Supplementary Concept] OR "bosutinib"[All Fields]) OR ("ponatinib"[Supplementary Concept] OR "ponatinib"[All Fields]) OR ("imatinib mesylate"[MeSH Terms] OR ("imatinib"[All Fields] AND "mesylate"[All Fields]) OR "imatinib mesylate"[All Fields] OR "imatinib"[All Fields] OR "imatinib s"[All Fields]) OR "TKI"[All Fields])) |

Supplementary table 2 Example of systematic search strategy in the present study
