# Supplementary material for: Arterial Hypertension and Tyrosine Kinase Inhibitors in Chronic Myeloid Leukemia: A Systematic Review and Meta-Analysis
Source: Front Pharmacol. 2021 Sep 22;12:674748. doi: 10.3389/fphar.2021.674748 (PMC8493251; doi:10.3389/fphar.2021.674748)
Supplement: Supplementary file 3 [file DataSheet1.docx]

Supplementary Figure S1 Pooled incidence rate of hypertension in patients treated with second or third generation TKI considering TKI-exposure time

Supplementary Figure S2 Pooled rate of hypertension when TKI were used in first or second line versus over second line considering TKI-exposure time
